# Risk of Sudden Sensorineural Hearing Loss in Patients with Gout: A Population-Level Study in a South Korean National Health Screening Cohort

**DOI:** 10.3390/jcm14041094

**Published:** 2025-02-08

**Authors:** Hyung-Bon Koo, Juyong Chung

**Affiliations:** Department of Otorhinolaryngology, Wonkwang University School of Medicine, 895 Muwang-ro, Iksan 54538, Republic of Korea; kgudqhs@naver.com

**Keywords:** gout, sudden sensorineural hearing loss, risk, comorbidity, population-level study

## Abstract

**Background**: Gout, characterized by serum uric acid accumulation, prompts inflammation, leading to tissue damage and comorbidities. Prior studies reported a higher risk of hearing loss in gout patients; however, the specific risk of sudden sensorineural hearing loss (SSNHL) remains unclear. **Method**: This population-based study assessed SSNHL incidence and risk in patients aged ≥ 40 years with or without gout, excluding those with prior SSNHL, within the Korean National Health Insurance Service Health Screening Cohort (2002–2019). A total of 24,508 gout patients were matched 1:4 with 98,032 controls by age, sex, income, and region. SSNHL incidence was compared, and Kaplan–Meier curves with log-rank tests evaluated cumulative incidence over 200 months. Hazard ratios (HRs) were calculated using stratified Cox models adjusted for patient characteristics. **Results**: SSNHL incidence was slightly higher in the gout cohort vs. controls (1.70% vs. 1.96%, SD = 0.02). Kaplan–Meier analysis revealed significantly higher cumulative SSNHL incidence in gout patients (*p* = 0.009). Patients with gout had a significantly higher risk of developing SSNHL in both the unadjusted (HR [95% CI]: 1.14 [1.03–1.27]; *p* = 0.010) and adjusted Cox models (1.13 [1.02–1.26]; *p* = 0.021). Subgroup analyses indicated higher risk in gout patients aged <60 years, males, non-smokers, non-drinkers, moderately-high income, normal BMI, Charlson Comorbidity Index score of 0, or fasting blood glucose < 100 mg/dL (all *p* < 0.05). **Conclusions**: Korean adults with gout, particularly younger, healthier patients, face increased SSNHL risk. Early, effective gout management may help mitigate this risk.

## 1. Introduction

Gout is the most common inflammatory arthritis, with a prevalence of <1–6.8% and incidence of 0.1–0.3% depending on the region, and has an overall increasing trend worldwide [1,2,3,4]. The disease is caused by persistent elevation of serum uric acid levels (hyperuricemia), leading to the supersaturation and deposition of urate crystals in joints, tendons, and other tissues [5]. Clinical symptoms include acute, recurrent pain, swelling, inflammation of affected joints, and, when chronic, arthropathy and negative impacts on organ function (i.e., nephropathy) [3]. Gout is typically diagnosed by measuring the levels of urate crystals in synovial fluid and blood, along with imaging to detect depositions (tophi) and assess inflammation [6,7]. Although there is no definitive cure for gout, medications that suppress inflammation and reduce hyperuricemia can prevent or even reverse the destructive disease pathology [8].

Various comorbid disorders can contribute to or result from gout, and there are numerous epidemiological studies reporting demographic, clinical, and other risk factors for the disease. Gout is generally more common among males than females, although the risk increases in females post-menopause [9]. The overall prevalence increases with each decade of life, up to 11–13% in people aged ≥ 80 years [2,3], but the mean age at onset is trending younger [10]. Other than hyperuricemia, established risk factors include hypertension, diuretic use, higher Body Mass Index (BMI), dietary factors (i.e., excessive consumption of red meat, seafood/shellfish, sugar, or alcohol), certain genetic variants, obstructive sleep apnea, diabetes, and renal, metabolic, and cardiovascular disease [2,11,12,13,14]. Additionally, the development of gout may increase the risk of other disorders such as heart attack or failure, stroke, erectile dysfunction, atrial fibrillation, osteoporosis and fracture, and venous thromboembolism [15,16,17,18,19].

Notably, there is an emerging body of literature reporting a greater risk of hearing loss among patients with gout or hyperuricemia [20,21,22,23,24,25], which has been variously attributed to tophi in the middle ear [26,27,28], exacerbation of age-related hearing loss [23], or inflammatory or oxidative damage to the sensory cells of the cochlea [24]. However, there remains limited evidence regarding the risk of sudden sensorineural hearing loss (SSNHL) among people with gout, particularly among Asian populations. SSNHL is an acute form of hearing loss characterized by a sudden decrease in hearing sensitivity of ≥30 decibels (dB) in three consecutive frequencies over three days [29,30]. The incidence of SSNHL has been estimated at 27 per 100,000 persons and increases with age, up to 77 per 100,000 people aged ≥ 65 years [31]. SSNHL is a challenging disorder to treat due to its sudden onset or unpredictable likelihood of resolution, and its etiology is not well established [32,33]. However, auto-immune dysfunction and inflammation are among the potential contributors to SSNHL [32,34], which are also present in gout [35,36], providing a rationale to investigate their co-occurrence. Further, the prevalence of gout in South Korea has dramatically increased over time, from 0.39% in 2002 to 2.01% in 2015 [37], emphasizing the importance of identifying associated risks of the disease to inform treatment plans.

Accordingly, this cross-sectional, population-based study evaluated the incidence and cumulative incidence of emergent SSNHL among adults with and without a diagnosis of gout using a large, nationwide healthcare database in South Korea. We compared the risk of developing SSNHL among adults with gout and matched controls, with or without adjustment for patient characteristics, among subgroups defined by demographic and clinical traits.

## 2. Materials and Methods

### 2.1. Data Source

This study used de-identified data from patients in the Korean National Health Insurance Service Health Screening Cohort (KNHISHSC). A detailed description of the KNHISHSC patient population has been previously published [38,39]. The KNHISHSC chooses a random 10% sample among adults age ≥ 40 and their families receiving healthcare, which between 2002 and 2019 included a total of 514,866 participants with 895,300,177 medical claim records. Data elements include demographics, socioeconomic data, health insurance claim codes (procedures and prescriptions), diagnostic codes using the International Classification of Disease-10 (ICD-10), death records, and health check-up data, including prescriptions and lab results (i.e., fasting glucose, total cholesterol, etc.) for each participant.

### 2.2. Ethics

The Ethics Committee of Hallym University in Chuncheon, South Korea, approved this study (Ethics Approval Code: 2019-10-023) on 23 October 2019. The requirement for written informed consent was waived by the Institutional Review Board. All analyses adhered to the guidelines and regulations of the Ethics Committee of Hallym University and the Declaration of Helsinki.

### 2.3. Participant Selection and Matching

**Figure 1:**
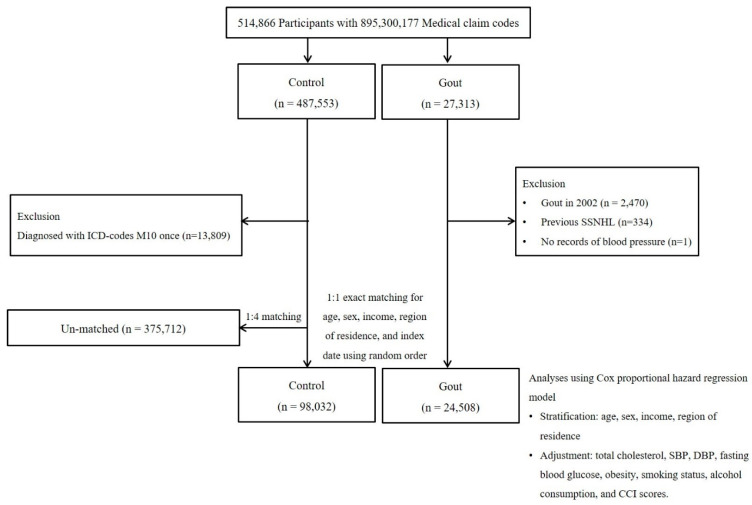
Study selection flowchart. Of 514,866 total patients in the database, 24,508 met all study criteria for inclusion into the gout cohort and were matched with 98,032 healthy controls on age, sex, income, and region of residence. Abbreviations: CCI, Charlson Comorbidity Index; DBP, diastolic blood pressure; ICD-10, International classification of disease-10; SBP, systolic blood pressure.

Figure 1 illustrates the selection steps for the study cohorts. Patients were eligible for inclusion in the gout cohort if they had (1) ≥2 healthcare visits with diagnoses of idiopathic gout (International Classification of Diseases, Tenth Revision (ICD-10) code M10) during 2002–2019; (2) no healthcare visit with a diagnosis of SSNHL (ICD-10 code H91.2) prior to the index date, defined as the date of the first diagnosis of gout; and (3) blood pressure measurements available in their records. Patients had to be ≥40 years old at the index date due to the criteria for selection into the KNHISHSC database. The method used to identify gout diagnoses has been previously published [40]. 

Patients without a diagnosis of gout at any time between 2002 and 2019 and who met all other criteria listed above were selected as potential controls. From a total of 514,866 individuals with 895,300,177 medical claim records spanning 2002 to 2019, 27,313 were identified as gout patients. Participants without a gout diagnosis at any time between 2002 and 2019 were assigned to the control group (*n* = 487,553). To ensure inclusion of only newly diagnosed gout cases, individuals with a gout diagnosis in 2002 were excluded from the cohort (washout period: *n* = 2470). Any individual with a prior diagnosis of sudden sensorineural hearing loss (SSNHL) before the index date was removed from both the gout and control cohorts. A total of 334 individuals were excluded from the gout cohort. One participant from the gout cohort was removed due to missing blood pressure data. Participants in the control group who had a single recorded diagnosis of gout (ICD-10 code M10) were excluded (*n* = 13,809).

Participants in the gout cohort were matched 1:4 with those in the control cohort on age, sex, income level, and region of residence. To prevent selection bias during matching, controls were sorted using a random number order and then selected from top to bottom for matching. It was assumed that the matched controls were being evaluated at the same time as each matched patient in the gout cohort (i.e., assigned the same index date). Therefore, controls who died before their assigned index date were excluded from the matching process. During the matching procedure, 375,712 control participants were excluded. Finally, 24,508 gout participants were matched at a ratio of 1:4 with 98,032 control participants (Figure 1).

### 2.4. Study Outcomes

The study outcomes included the incidence of newly emerging SSNHL during the study period, defined as the time from the index date to 31 December 2019. SSNHL was identified in the claims as visits with ICD-10 code H91.2 among participants who underwent audiometric examination (claim code: E6931-E6937, F6341-F6348) and used steroids for treatment, as described in Choi et al. [41].

Demographic (age, sex, income level, and region) and clinical characteristics at the index date were recorded for both cohorts as potential covariates. The region of residence was grouped into urban (Seoul, Busan, Daegu, Incheon, Gwangju, Daejeon, and Ulsan) and rural (Gyeonggi, Gangwon, Chungcheongbuk, Chungcheongnam, Jeollabuk, Jeollanam, Gyeongsangbuk, Gyeongsangnam, and Jeju) areas. Ten age groups were specified using 5-year intervals starting from age 40 and extending beyond 85 years. Additionally, income was categorized into five groups, with class 1 representing the lowest (0–20%) and class 5 the highest (81–100%). Clinical characteristics included smoking status (non-smoker, past smoker, or current smoker), alcohol consumption frequency (<1 or ≥1 time per week), body mass index (BMI, kg/m^2^), blood pressure (systolic and diastolic, mmHg), fasting blood glucose levels (mg/dL), and total serum cholesterol (mg/dL). According to the Asia-Pacific criteria of the World Health Organization [42], BMI classifications included underweight (<18.5), normal (≥18.5 to <23), overweight (≥23 to <25), obese I (≥25 to <30), and obese II (≥30). Charlson Comorbidity Index (CCI) score was computed for each participant according to the severity and number of diseases listed in the CCI, with potential scores ranging from 0 (no comorbidities) to 29 (highest severity of all comorbidities) as described by Quan et al. [43,44].

### 2.5. Data Analysis

The standardized difference (Std Diff) was used to assess differences in baseline characteristics of the gout cohort and matched controls after matching. A Kaplan–Meier estimator, along with a log-rank test, was utilized to determine the cumulative occurrence of SSNHL among the gout and matched control cohorts over up to 200 months.

Both unadjusted and adjusted stratified Cox proportional hazards models were applied to evaluate the risk of SSNHL, with results presented as hazard ratios (HRs) and corresponding 95% confidence intervals (CIs). The adjusted models considered the covariates of BMI, smoking status, alcohol intake frequency, blood pressure levels, glucose metabolism, lipid profile, and overall comorbidity burden. The analysis was stratified based on demographic factors, including age category, gender, economic status, and residential area (urban vs. rural). For subgroup analyses with the stratified Cox proportional hazards model, the cohorts were categorized based on age (<60 vs. ≥60 years), biological sex, BMI category, and fasting glucose levels (<100 vs. ≥100 mg/dL). In the unstratified Cox proportional hazards model, subgroup analyses classified participants by income level, residential area (urban vs. rural), smoking behavior; alcohol intake, systolic blood pressure (<120 mmHg, ≥120 mmHg and <140 mmHg, ≥140 mmHg), diastolic blood pressure (<80 mmHg, ≥80 mmHg and <90 mmHg, ≥90 mmHg), total cholesterol (<200 mg/dL, ≥200 mg/dL and <240 mmHg, ≥240 mmHg), and CCI score (0, 1, and ≥2).

Two-tailed analyses were performed, and significance was defined as *p*-values of less than 0.05. All analyses were conducted using SAS version 9.4 (SAS Institute Inc., Cary, NC, USA).

## 3. Results

### 3.1. Cohort Characteristics

A total of 24,508 patients met all criteria for inclusion in the gout cohort and were matched with 98,032 controls (Figure 1). The demographic and clinical profiles of the gout cohort and matched controls are summarized in Table 1. After matching, both cohorts had comparable distributions for the matched-on characteristics of age, sex, income level, and region of residence (all Std Diff: 0.0). In the study population, 78.85% were male, 36.79% belonged to income level 5, and 57.79% resided in rural areas. Additionally, variations between the cohorts were minimal across BMI category, smoking habits, alcohol intake, fasting glucose, blood pressure (systolic/diastolic), total cholesterol, and CCI score. Compared to controls, there was a higher proportion of patients in the gout cohort in the BMI category of obese I (41.65% vs. 32.14% (control)) and a lower proportion with normal BMI (25.08% vs. 34.82%).

### 3.2. Overall Incidence of SSNHL Among Patients with or Without Gout

**Figure 2:**
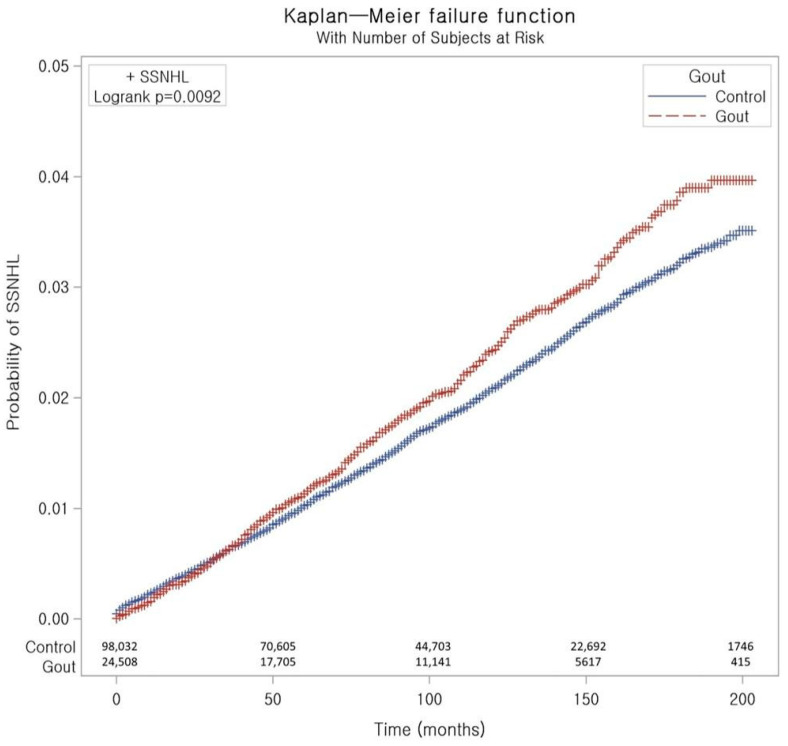
Kaplan–Meier failure curve and cumulative incidence function for the occurrence of SSNHL in patients with and without gout. The number at risk over time is shown at the bottom of the figure. Abbreviation: SSNHL, sudden sensorineural hearing loss.

Of those who developed SSNHL, 1.94% (476/24,508) were in the gout cohort, and 1.70% (1663/98,032) were in the control cohort (Std Diff: 0.02, Table 1). The incidence of SSNHL was higher in the gout cohort than in the matched control cohort (2.6 vs. 2.3 per 10,000 person-years; Table 2). The Kaplan–Meier failure curve and cumulative incidence function for the occurrence of SSNHL during the up to 200-month follow-up period are presented in Figure 2. The log-rank test indicated that patients with gout experienced a significantly higher cumulative incidence of SSNHL than the control cohort during follow-up (*p* = 0.009).

### 3.3. Risk of SSNHL Among Patients with or Without Gout, Overall and Among Subgroups

In Table 2, the HRs for SSNHL are listed for the gout and control cohorts overall and categorized by age, sex, weight, BMI category, and fasting blood glucose levels. During the study period, patients in the gout cohort had a higher risk of SSNHL, with a 1.14-fold increase (95% CI: 1.03–1.27, *p* = 0.010) in the unadjusted model and a 1.13-fold increase (95% CI: 1.02–1.26, *p* = 0.021) after adjustment for covariates. In the subgroup analyses, the higher risk of SSNHL among patients with gout persisted among those who had normal BMI, were aged < 60 years, were male, and had fasting blood glucose < 100 mg/dL in both unadjusted and adjusted models (all *p* < 0.05 vs. controls).

In Table 3, the results showed a significantly greater risk of SSNHL among patients with gout who were in income level 4, were nonsmokers, consumed alcohol less than once per week, and had a CCI score of 0 in both the unadjusted and adjusted models (all *p* < 0.05 vs. controls). Additionally, patients with gout living in rural regions or with systolic blood pressure < 120 mmHg had a significantly higher risk of SSNHL in the unadjusted model only (*p* < 0.05 vs. controls).

## 4. Discussion

Reflecting global trends in recent decades [45], the incidence and prevalence of gout in Asian nations have increased. In South Korea, gout prevalence has risen from 0.35% in 2007 to 0.76% in 2015 and is expected to be 1.66% by 2025 [46]. Many potential complications have been associated with gout, including hearing loss. Therefore, understanding the associations between gout and SSNHL is important for reducing the burden of both diseases. This cross-sectional, population-level study of South Korean patients in a national health database is the first, to our knowledge, to assess the epidemiological trends and risk of SSNHL in patients with gout. We observed a higher incidence of SSNHL among patients with gout compared to matched controls without the disease (2.60 vs. 2.27 per 10,000 person-years, respectively), as well as higher prevalence (1.94% vs. 1.70%) over the 2002–2019 period. Additionally, a significantly elevated risk of SSNHL was observed for patients with gout compared to matched controls, even after adjusting for patient characteristics, overall and among subgroups defined by demographic and clinical features.

The overall incidence of SSNHL in this study, both among those with and without gout, is slightly higher than the first estimates of unilateral SSNHL in South Korea using Health Insurance Review and Assessment Service data from 2011 to 2015 (1.78 cases/10,000 people) [47]. That study also observed that unilateral SSNHL was more common among females compared to males and increased in incidence by decades of life until age 70 years, when it then sharply declined [47]. These prior findings help provide context for the impact of gout on the risk of SSNHL, as we observed significantly higher risk among males and younger (aged < 60 years) people with gout compared to matched controls. Additionally, a significantly higher risk of SSNHL was observed in generally healthier patients with gout, including those with normal weight, lower blood glucose levels, medium-high income (level 4), non-smokers, non-drinkers, and those with a CCI score of 0.

The overall prevalence of gout is substantially higher among males than females worldwide, from three to ten times more common depending on the region [3,48], which may potentially be related to better estrogen-mediated uric acid excretion among premenopausal women [9]. In the Korean population, the prevalence of gout was previously reported to be >5:1 in males versus females (2009–2015) [40]. Our population with gout was comprised of 78% males and 21% females. The overall incidence of SSNHL was numerically higher among females than males with gout (2.70 vs. 2.57 per 10,000 person-years); however, only males with gout showed a significantly higher risk of SSNHL compared to matched controls.

The majority of SSNHL cases are unilateral and idiopathic, with wide distributions by age and no established sex preference [29,49]. The underlying causes of SSNHL remain unclear, potentially involving vascular abnormalities, infections, autoimmune reactions, ototoxicity, trauma, tumors, and metabolic disorders, although ~90% of cases have unknown etiology [29,34,49]. Known otologic causes include endolymphatic hydrops (a feature of Meniere’s disease) and vestibular schwannoma [49]. However, there is conflicting evidence as to associated risk factors that overlap with gout, and hypertension, diabetes, and alcohol or tobacco use were found to be unrelated to SSNHL in a recent prospective cohort study among Asian patients [50]. Notably, the differentially higher risk of SSNHL in patients with versus without gout in this study was greatly delayed after initial gout diagnosis (i.e., the index date), only appearing years later (i.e., 50+ months). This risk difference persisted and increased over 200 months of follow-up.

This presents the question as to whether the etiology of SSNHL in gouty patients is related to primary disease pathology impacting the inner ear or other factors, such as ototoxicity from the medications used to treat and prevent gout. Some studies have reported that patients with hyperuricemia or gout have reduced (worse) transient evoked and distortion product otoacoustic emissions, a measure of outer hair cell function, or abnormal audiometry [51,52], while others have observed no or only higher frequency-specific differences [22,24]. Paradoxically, uric acid is an antioxidant [53], and higher serum uric acid levels were observed to be protective of hearing threshold shift (i.e., hearing loss) in a nationally representative sample of adults in the United States (US) [54]. Additionally, a study in a Turkish population reported no association between mean uric acid levels and incidence of SSNHL specifically, and the post-treatment pure-tone average was significantly better in patients with higher serum uric acid levels [55]. Conversely, a case-control study of Italian patients with SSNHL observed that higher uric acid levels, along with blood glucose and coagulative factors, were positively correlated with worse SSNHL severity [56]. At the population level, a retrospective study observed that gout increased the risk of new hearing loss by 44% in US patients aged ≥ 65 years (2006–2012), even after adjusting for clinical characteristics and gout medication use [20]. Similarly, hyperuricemia was independently associated with hearing impairment in South Korean patients, particularly among females, those with prior noise exposure, and people aged ≥ 60 years [25]. However, the patients at risk in the prior two studies contrast with those of the current study (i.e., males and age < 60 years) and experienced any form of hearing loss (not just SSNHL). The causes and natural history of different forms of hearing loss differ, and the risk of any hearing loss (including age-related or noise-induced) may not be the same as that of SSNHL. It is also important to note that while hyperuricemia is the cause of gout, most patients with hyperuricemia will not develop gout, and therefore, the relationship between the two disorders and SSNHL may differ.

Although the pathological mechanisms of SSNHL are yet unknown, gout may negatively impact the inner ear via the generation of reactive oxygen species (ROS) as a consequence of hyperuricemia and urate crystal deposition [57,58]. In support of this, hyperuricemia may contribute to hearing impairment through several interrelated mechanisms that compromise cochlear function. One plausible mechanism involves the adverse effects of accumulated uric acid on the vascular endothelium. Uric acid can impair endothelial function by reducing the bioavailability of nitric oxide and inhibiting endothelial cell proliferation, which results in compromised blood flow [59,60]. In parallel, hyperuricemia is associated with enhanced platelet activation, further promoting endothelial dysfunction. Given that the cochlea receives its blood supply from a single arterial source [61], even subtle disturbances in microcirculation may lead to significant subclinical damage and ultimately contribute to sensorineural hearing loss. Furthermore, a reduction in blood supply to the inner ear has been implicated in the development of SSNHL as well as other otologic conditions, such as Meniere’s disease and vestibular neuritis [62,63]. Another important mechanism is uric acid-induced systemic inflammation. Elevated serum uric acid levels have been linked to higher concentrations of inflammatory markers, including C-reactive protein, IL-6, IL-18, and TNF-α, as observed in studies among older adults [64,65]. This inflammatory response may be amplified locally in the inner ear by the formation of monosodium urate crystals, which activate toll-like receptors and trigger the NALP3 inflammasome. This cascade promotes the release of pro-inflammatory cytokines such as IL-1, further fueling an inflammatory milieu that damages cochlear cells [66,67]. Pro-inflammatory mediators associated with gout, such as IL-1 and IL-6, can directly damage inner ear cells [68,69,70]. Experimental studies have demonstrated that elevated levels of these cytokines in the cochlea are associated with leukocyte infiltration, scar formation, and gliosis—processes linked to immune-mediated hearing loss [71,72]. Additionally, uric acid can act as a pro-oxidant by stimulating the production of ROS in vascular cells [73]. The resulting overproduction of ROS may overwhelm the cochlea’s endogenous antioxidant systems, leading to lipid peroxidation and cellular injury. Excessive ROS levels have been implicated in damage to the cochlear sensory epithelium, spiral ganglion neurons, and the stria vascularis, all of which are key factors in the development of sensorineural hearing loss [74,75,76,77]. Although uric acid also possesses antioxidant properties under normal conditions [78,79], sustained hyperuricemia may tip the balance in favor of oxidative stress, thereby exacerbating cellular damage within the cochlea. Moreover, other clinical features that impact microcirculation—such as hypertension, hyperlipidemia, gluco-metabolic disorders, and coagulative factors—are associated with SSNHL etiology and its severity in some studies [56,80]. In a cohort of patients with gout aged > 50 years in the United Kingdom, there was a significantly higher risk of peripheral vascular disease within 10 years following gout onset, with females at the highest risk [81].

Recent evidence suggests that hyperuricemia and gout may not only be risk factors for the development of SSNHL but also serve as independent prognostic indicators for hearing recovery. In a recent retrospective study, elevated serum uric acid levels and hyperuricemia were significantly associated with poorer hearing outcomes in SSNHL patients [82]. Uric acid, the final product of purine metabolism, is closely linked to vascular endothelial function and oxidative stress injury [83]. Experimental studies, both in vitro and in vivo, have demonstrated that increased uric acid levels can exacerbate oxidative stress, accelerate immunosenescence, and initiate inflammatory processes in inner hair cells, thereby contributing to cochlear degeneration and sensory impairment [22,84]. Furthermore, patients with hyperuricemia exhibit a greater propensity for platelet dysfunction, thrombosis, and overall endothelial dysregulation, which may elevate the risk of acute vascular events and ultimately lead to a poorer clinical prognosis [85,86,87]. These findings support the hypothesis that patients with gout, a condition inherently associated with hyperuricemia, may experience less favorable recovery compared to those with viral-associated (neuritis) SSNHL due to underlying microangiopathic changes and acute vascular events affecting the inner ear. Although our registry did not allow for direct evaluation of these associations, these insights underscore the potential prognostic impact of hyperuricemia and gout on SSNHL recovery and highlight the need for further investigation.

Given the potential impact of hyperuricemia and vascular dysfunction on SSNHL recovery, optimizing treatment strategies is essential. Standard therapeutic approaches aim to restore hearing by addressing inflammation, vascular insufficiency, and immune responses. The first-line treatment for SSNHL is systemic corticosteroid therapy, typically administered orally or intravenously, due to its anti-inflammatory and immunosuppressive effects [30]. However, intratympanic corticosteroid (ITC) therapy has gained increasing recognition, particularly in patients with contraindications to systemic steroids or those with persistent hearing loss despite initial treatment [31]. Among intratympanic steroids, Triamcinolone Acetonide (ITA) has shown a more appropriate pharmacokinetic profile for local therapy of hearing disorders, with a longer half-life in the cochlea compared to dexamethasone [88]. These properties make ITA a suitable option for SSNHL salvage therapy after the failure of first-line treatment [88]. Other treatment options include hyperbaric oxygen therapy (HBOT), which enhances cochlear oxygenation [89], and vasodilators, which aim to improve inner ear microcirculation. While antiviral agents and plasma expanders have been explored, their efficacy remains uncertain [90].

Another important consideration is that medications used to manage gout may also affect auditory function. Given the substantial delay in SSNHL risk observed in this study (i.e., around 50 months post-gout diagnosis), it is also possible that medications used to control gout flares and reduce serum urate may have ototoxic effects over time. The most common initial treatment for gout in South Korea (as reported in 2017) was allopurinol (83.8%): 62.3% also took colchicine, and 39.9% took non-steroidal anti-inflammatory inhibitor drugs (NSAIDs) in combination with primary treatment [91]. Additionally, NSAIDs are used for long-term gout prophylaxis as per Korean clinical treatment guidelines [92]. Hearing loss is not a common complication with allopurinol, yet has been noted [93]. In contrast, studies in animal models have observed a protective effect of allopurinol for noise-induced or cisplatin-related hearing loss [94,95,96], which may be related to the reduction in oxidative stress by lowering serum uric acid levels [97]. However, the use of NSAIDs can lead to hearing loss and SSNHL, especially in men and younger people, and the risk increases with the duration of use and dosing [98,99]. NSAIDs have been shown to be ototoxic in animal models and in humans, potentially through a reduction in cochlear blood flow [99,100,101]. Therefore, cautious use and dosing of NSAIDs may be recommended to lower the risk of SSNHL in patients with gout.

This study benefits from several strengths. We used a large study population and implemented measures to minimize bias. The large population-based cohort study provided data on the temporal relationship between the initial diagnosis of gout and the occurrence of SSNHL. Additionally, we considered demographic, socioeconomic, and lifestyle, in addition to certain comorbid conditions in the adjusted analyses.

This study is also subject to several limitations, some of which are inherent to cross-sectional studies using population-level registry data (i.e., missing data, miscoding, etc.). First, although diagnostic codes were used to identify SSNHL, the database used did not allow for the evaluation of the type or degree of SSNHL. Second, we did not analyze comorbid conditions such as cerebrovascular disease and cardiovascular disease, which could affect SSNHL risk; however, as these conditions are part of the CCI score, our adjustment for the CCI score should address this limitation to some extent. Despite adjusting for many variables, unmeasured confounding factors such as stress, nutritional status, and physical activity, which were not available in our dataset, may have influenced the results. Third, the use of gout medication, including adherence or non-adherence to prescribed regimens, was not assessed in this study. Finally, the pathophysiological mechanisms underlying the relationship between gout and SSNHL are not fully understood, and further research is needed to explore how controlling gout may impact the presence of SSNHL.

## 5. Conclusions

This cross-sectional, population-level study of Korean patients found an elevated risk of SSNHL among patients with gout compared to those without the disease, including after adjusting for various demographic and clinical characteristics. These findings highlight the need for further investigation into the pathophysiological mechanisms connecting gout, hyperuricemia, and auditory function to increase our understanding of the risk of SSNHL in this patient population. Early diagnosis and effective management of gout, as well as audiological monitoring during treatment, may reduce the risk of developing comorbid SSNHL.

## Figures and Tables

**Table 1:** Cohort Demographic and Clinical Characteristics After Matching ^a^.

	Total Participants, *n* (%)		
	Gout Cohort N = 24,508	Control Cohort N = 98,032	Standardized Difference
**Demographic Characteristics**			
Age (years)			0.00
40–44	592 (2.42)	2368 (2.42)	
45–49	2089 (8.52)	8356 (8.52)	
50–54	3652 (14.90)	14,608 (14.90)	
55–59	4790 (19.54)	19,160 (19.54)	
60–64	4178 (17.05)	16,712 (17.05)	
65–69	3504 (14.30)	14,016 (14.30)	
70–74	2756 (11.25)	11,024 (11.25)	
75–79	1789 (7.30)	7156 (7.30)	
80–84	875 (3.57)	3500 (3.57)	
85+	283 (1.15)	1132 (1.15)	
Sex			0.00
Male	19,327 (78.86)	77,308 (78.86)	
Female	5181 (21.14)	20,724 (21.14)	
Income			0.00
1 (lowest)	3579 (14.60)	14,316 (14.60)	
2	3011 (12.29)	12,044 (12.29)	
3	3728 (15.21)	14,912 (15.21)	
4	5174 (21.11)	20,696 (21.11)	
5 (highest)	9016 (36.79)	36,064 (36.79)	
Region of residence			0.00
Urban	10,345 (42.21)	41,380 (42.21)	
Rural	14,163 (57.79)	56,652 (57.79)	
**Clinical characteristics**			
BMI category ^b^			0.27
Underweight	332 (1.35)	2421 (2.47)	
Normal	6146 (25.08)	34,131 (34.82)	
Overweight	6733 (27.47)	27,461 (28.01)	
Obese I	10,208 (41.65)	31,512 (32.14)	
Obese II	1089 (4.44)	2507 (2.56)	
Smoking status			0.07
Nonsmoker	13,263 (54.12)	52,575 (53.63)	
Past smoker	5796 (23.65)	20,956 (21.38)	
Current smoker	5449 (22.23)	24,501 (24.99)	
Alcohol consumption			0.09
<1 time a week	11,922 (48.65)	52,313 (53.36)	
≥1 time a week	12,586 (51.35)	45,719 (46.64)	
Systolic blood pressure, mmHg			0.13
<120	5898 (24.07)	27,371 (27.92)	
120–139	12,887 (52.58)	49,537 (50.53)	
≥140	5723 (23.35)	21,124 (21.55)	
Diastolic blood pressure, mmHg			0.10
<80	12,927 (52.75)	44,186 (45.07)	
80–89	8342 (34.04)	36,290 (37.02)	
≥90	3239 (13.22)	17,556 (17.91)	
Fasting blood glucose, mg/dL			0.01
<100	13,811 (56.35)	56,840 (57.98)	
100–125	10,697 (43.65)	41,192 (42.02)	
≥126	13,811 (56.35)	56,840 (57.98)	
Total cholesterol, mg/dL			0.08
<200	13,188 (53.81)	55,758 (56.88)	
200–239	7820 (31.91)	30,784 (31.40)	
≥240	3500 (14.28)	11,490 (11.72)	
CCI score			0.15
0	13,142 (53.62)	58,989 (60.17)	
1	4267 (17.41)	15,668 (15.98)	
≥2	7099 (28.97)	23,375 (23.84)	
SSNHL	476 (1.94)	1663 (1.70)	0.02

Abbreviations: BMI, Body Mass Index; CCI, Charlson comorbidity index; SSNHL, sudden sensorineural hearing loss. Note: ^a^ Patients in the gout cohort were matched to controls according to age, sex, income level, and region of residence. ^b^ BMI (kg/m^2^) was categorized as <18.5 (underweight), ≥18.5 to <23 (normal), ≥23 to <25 (overweight), ≥25 to <30 (obese I), and ≥30 (obese II).

**Table 2:** Unadjusted and adjusted risk of SSNHL among patients with and without gout, overall, by age, sex, BMI, and fasting blood glucose level.

	IR per 10,000 Person-Years	IRD per 10,000 Person-Years (95% CI)	HR for SSNHL (95% CI)			
			Unadjusted ^a^	*p*	Adjusted ^a,b^	*p*
**Total participants (N = 122,540)**						
Gout	2.60	3.29 (0.82 to 5.76)	1.14 (1.03–1.27)	0.010 *	1.13 (1.02–1.26)	0.021 *
Control	2.27		1		1	
**Age**						
<60 years (*n* = 55,615)						
Gout	2.52	3.53 (0.80 to 6.27)	1.19 (1.03–1.36)	0.015 *	1.18 (1.02–1.36)	0.022 *
Control	2.13		1		1	
≥60 years (*n* = 66,925)						
Gout	2.69	2.34 (−3.42 to 8.10)	1.10 (0.94–1.28)	0.233	1.07 (0.92–1.25)	0.372
Control	2.45		1		1	
**Sex**						
Men (*n* = 96,635)						
Gout	2.57	3.95 (0.77 to 7.14)	1.16 (1.03–1.30)	0.012 *	1.13 (1.00–1.27)	0.047 *
Control	2.22		1		1	
Women (*n* = 25,905)						
Gout	2.70	2.41 (−1.49 to 6.31)	1.09 (0.87–1.37)	0.440	1.13 (0.90–1.41)	0.302
Control	2.47		1		1	
**BMI category**						
Underweight (*n* = 2753)						
Gout	2.97	15.05 (−3.74 to 33.84)	2.66 (0.94–7.51)	0.065	2.84 (0.98–8.19)	0.054
Control	1.47		1		1	
Normal weight (*n* = 40,277)						
Gout	2.86	8.04 (3.33 to 12.75)	1.39 (1.14–1.69)	0.001 *	1.39 (1.14–1.70)	0.001 *
Control	2.06		1		1	
Overweight (*n* = 34,194)						
Gout	2.51	−0.90 (−5.83 to 4.03)	0.99 (0.81–1.20)	0.887	1.01 (0.83–1.23)	0.958
Control	2.60		1		1	
Obese I (*n* = 41,720)						
Gout	2.41	1.54 (−2.32 to 5.41)	1.08 (0.91–1.27)	0.384	1.06 (0.90–1.26)	0.473
Control	2.26		1		1	
Obese II (*n* = 3596)						
Gout	3.42	12.42 (−1.12 to 25.95)	1.54 (0.92–2.59)	0.103	1.44 (0.84–2.46)	0.183
Control	2.18		1		1	
**Fasting blood glucose**						
<100 mg/dL (*n* = 70,651)						
Gout	2.62	4.17 (1.02 to 7.33)	1.19 (1.04–1.36)	0.010 *	1.17 (1.03–1.34)	0.020 *
Control	2.21		1		1	
≥100 mg/dL (*n* = 51,889)						
Gout	2.55	1.90 (−2.08 to 5.88)	1.08 (0.92–1.27)	0.372	1.06 (0.90–1.25)	0.513
Control	2.36		1		1	

Abbreviations: BMI, Body Mass Index; IR, incidence rate; IRD, incidence rate difference; SSNHL, sudden sensorineural hearing loss. Notes: ^a^ Models were stratified by age, sex, income, and region of residence. ^b^ The model was adjusted for BMI score, smoking, alcohol consumption, systolic blood pressure, diastolic blood pressure, fasting blood glucose, total cholesterol, and Charlson Comorbidity Index score. * *p* < 0.05.

**Table 3:** The unadjusted and adjusted risk of SSNHL among patients with and without gout, overall, by income level, region, smoking status, alcohol consumption, blood pressure, fasting blood glucose, total cholesterol, and CCI score.

	IR per 10,000 Person-Year	IRD per 10,000 Person-Years (95% CI)	HRs for SSNHL (95% CI)			
			Unadjusted ^a^	*p*	Adjusted ^a,b^	*p*
**Income level**						
Level 1 (lowest) (*n* = 17,895)						
Gout	2.70	6.33 (−0.25 to 12.91)	1.31 (0.99–1.73)	0.058	1.32 (0.99–1.76)	0.055
Control	2.07		1		1	
Level 2 (*n* = 15,055)						
Gout	2.18	−1.10 (−8.13 to 5.93)	0.96 (0.70–1.31)	0.775	0.93 (0.67–1.27)	0.632
Control	2.29		1		1	
Level 3 (*n* = 18,640)						
Gout	2.55	3.92 (−2.29 to 10.12)	1.18 (0.90–1.53)	0.231	1.13 (0.86–1.48)	0.398
Control	2.16		1		1	
Level 4 (*n* = 25,870)						
Gout	2.82	6.06 (0.70 to 11.43)	1.27 (1.02–1.57)	0.031 *	1.30 (1.04–1.62)	0.022 *
Control	2.21		1		1	
Level 5 (highest) (*n* = 45,080)						
Gout	2.58	1.83 (−2.25 to 5.91)	1.08 (0.91–1.27)	0.378	1.06 (0.89–1.25)	0.523
Control	2.40		1		1	
**Region of residence**						
Urban residents (*n* = 51,725)						
Gout	2.52	3.05 (−0.67 to 6.77)	1.14 (0.97–1.33)	0.112	1.12 (0.95–1.32)	0.164
Control	2.21		1		1	
Rural residents (*n* = 70,815)						
Gout	2.65	3.47 (0.16 to 6.78)	1.15 (1.01–1.31)	0.041 *	1.14 (0.99–1.30)	0.069
Control	2.31		1		1	
**Smoking status**						
Nonsmoker (*n* = 65,838)						
Gout	2.88	4.35 (0.89 to 7.81)	1.18 (1.04–1.34)	0.013 *	1.20 (1.05–1.37)	0.008 *
Control	2.45		1		1	
Past smoker (*n* = 26,752)						
Gout	2.53	0.41 (−5.46 to 6.27)	1.01 (0.80–1.28)	0.910	0.99 (0.78–1.26)	0.921
Control	2.49		1		1	
Current smoker (*n* = 29,950)						
Gout	2.00	2.31 (−2.02 to 6.64)	1.12 (0.89–1.42)	0.322	1.07 (0.85–1.36)	0.558
Control	1.77		1		1	
**Alcohol consumption**						
<1 time a week (*n* = 64,235)						
Gout	2.88	5.86 (2.42 to 9.29)	1.25 (1.09–1.43)	0.001 *	1.26 (1.10–1.44)	0.001 *
Control	2.30		1		1	
≥1 time a week (*n* = 58,305)						
Gout	2.28	0.56 (−3.00 to 4.11)	1.03 (0.88–1.21)	0.719	0.98 (0.84–1.16)	0.836
Control	2.22		1		1	
**Blood pressure**						
SBP < 120 mmHg (*n* = 33,269)						
Gout	2.96	5.47 (0.30 to 10.63)	1.23 (1.01–1.50)	0.036 *	1.18 (0.97–1.44)	0.099
Control	2.41		1		1	
120 mmHg ≤ SBP < 140 mmHg (*n* = 62,424)						
Gout	2.55	2.84 (−0.57 to 6.25)	1.13 (0.98–1.30)	0.100	1.11 (0.96–1.29)	0.158
Control	2.26		1		1	
SBP ≥ 140 mmHg (*n* = 26,847)						
Gout	2.33	2.33 (−2.62 to 7.29)	1.09 (0.87–1.37)	0.439	1.08 (0.86–1.37)	0.503
Control	2.10		1		1	
DBP < 80 mmHg (*n* = 57,113)						
Gout	2.71	3.59 (0.04 to 7.14)	1.16 (1.00–1.33)	0.044 *	1.15 (1.00–1.34)	0.057
Control	2.35		1		1	
80 mmHg ≤ DBP < 90 mmHg (*n* = 44,632)						
Gout	2.44	1.37 (−2.84 to 5.57)	1.07 (0.89–1.27)	0.484	1.05 (0.88–1.26)	0.591
Control	2.31		1		1	
DBP ≥ 90 mmHg (*n* = 20,795)						
Gout	2.52	5.00 (−1.30 to 11.29)	1.25 (0.94–1.65)	0.122	1.29 (0.97–1.71)	0.078
Control	2.03		1		1	
**Cholesterol**						
Total cholesterol < 200 mg/dL (*n* = 68,946)						
Gout	2.58	2.54 (−0.92 to 6.01)	1.11 (0.96–1.28)	0.160	1.09 (0.94–1.26)	0.259
Control	2.33		1		1	
200 mg/dL ≤ Total cholesterol < 240 mg/dL (*n* = 38,604)						
Gout	2.57	3.33 (−0.93 to 7.59)	1.14 (0.95–1.36)	0.151	1.12 (0.93–1.34)	0.233
Control	2.24		1		1	
Total cholesterol ≥ 240 mg/dL (*n* = 14,990)						
Gout	2.70	6.22 (−0.05 to 12.49)	1.29 (0.98–1.68)	0.066	1.31 (0.99–1.72)	0.058
Control	2.07		1		1	
**CCI score**						
0 (*n* = 72,131)						
Gout	2.49	3.60 (0.38 to 6.83)	1.16 (1.01–1.34)	0.034 *	1.17 (1.01–1.35)	0.031 *
Control	2.13		1		1	
1 (*n* = 19,935)						
Gout	2.78	1.87 (−4.45 to 8.18)	1.05 (0.83–1.33)	0.694	1.03 (0.81–1.32)	0.806
Control	2.59		1		1	
≥2 (*n* = 30,474)						
Gout	2.68	2.74 (−2.17 to 7.64)	1.11 (0.91–1.35)	0.291	1.07 (0.88–1.30)	0.516
Control	2.40		1		1	

Abbreviation: CCI, Charlson Comorbidity Index; IR, incidence rate; IRD, incidence rate difference; SBP, systolic blood pressure; DBP, diastolic blood pressure; SSNHL, sudden sensorineural hearing loss. Notes: ^a,b^ See Table 2 for definitions. * *p* < 0.05.

## Data Availability

Releasing of the data by the researcher is not legally permitted. All data are available from the database of the Korea Center for Disease Control and Prevention. The Korea Center for Disease Control and Prevention allows data access, at a particular cost, for any researcher who promises to follow the research ethics. The data of this article can be downloaded from the website after agreeing to follow the research ethics.
